# Circumferential tracheal replacement with silicone stent supported, cryopreserved aortic homograft

**DOI:** 10.1093/jscr/rjae040

**Published:** 2024-02-09

**Authors:** Edward Hauptmann, Shumon Dhar, Omar Harirah, Raghav Chandra, Scott Reznik, John Waters

**Affiliations:** Department of Surgery, University of Texas Southwestern Medical Center, 6201 Harry Hines Blvd, Dallas, TX 75235, United States; Department of Otorhinolaryngology, University of Texas Southwestern Medical Center, 6201 Harry Hines Blvd, Dallas, TX 75235, United States; Department of Surgery, University of Texas Southwestern Medical Center, 6201 Harry Hines Blvd, Dallas, TX 75235, United States; Department of Surgery, University of Texas Southwestern Medical Center, 6201 Harry Hines Blvd, Dallas, TX 75235, United States; Division of Thoracic Surgery, Department of Cardiovascular and Thoracic Surgery, University of Texas Southwestern Medical Center, 6201 Harry Hines Blvd, Dallas, TX 75235, United States; Division of Thoracic Surgery, Department of Cardiovascular and Thoracic Surgery, University of Texas Southwestern Medical Center, 6201 Harry Hines Blvd, Dallas, TX 75235, United States

**Keywords:** tracheal replacement, aortic allograft, tracheal dehiscence

## Abstract

Treatment of long-segment tracheal defects remains a challenge in thoracic surgery with no standard surgical option. Aortic allografts have been used for this purpose with varying degrees of success. In a patient that suffered anastomotic dehiscence after tracheal resection with primary anastomosis, we performed complete tracheal resection and replacement using a stented circumferential aortic allograft. Currently, this patient is able to breathe normally without tracheostomy assistance 22 months postoperatively. Our report is the first in the English literature of long-term survival without tracheostomy dependence and close interval follow-up after circumferential tracheal resection and replacement with a cryopreserved aortic allograft.

## Introduction

Tracheal resection and primary anastomosis can be performed if <5 cm of the native trachea is removed [1]. Longer segment resections may require tracheal replacement [2]. The trachea is a dynamic component of air conduction with lateral rigidity, longitudinal flexibility, mucociliary clearance capabilities; it is intricately involved in chest and neck aerodigestive mechanics and cardiopulmonary physiology [3, 4]. Replacement matrices must be nontoxic, biocompatible, and able to epithelialize [4]. We present a case of circumferential tracheal replacement with cryopreserved aortic homograft as a salvage maneuver for tracheal anastomotic dehiscence following tracheal resection in a morbidly obese patient. This is the first case described of circumferential tracheal replacement with aortic homograft in a human adult in the English literature with long-term survival and close follow-up.

## Case report

A 17-year-old female (BMI 41) presented with tracheal stenosis following intubation after attempted suicide. The patient underwent previous bronchoscopic dilations prior to her hospitalization with minimal improvement. She was hospitalized for shortness of breath, requiring continuous supplemental oxygen support. During bronchoscopy, a stenotic tracheal segment 3.5 cm in length was noted 2 cm inferior to the cricoid cartilage, 4 cm superior to the carina with tracheomalacia distal to the stenotic segment. Treatment options were discussed with the patient and her family. Due to the patient’s psychiatric disease, there was concern about her ability to tolerate a long-term tracheostomy, T-tube, or maintain an airway stent. Furthermore, proceeding with either of these interventions would make a future resection more difficult. We decided that medical optimization and tracheal resection would be the best option. Psychiatry evaluated the patient, deeming her psychiatrically fit for surgery. Unfortunately, the patient developed acute hypoxic respiratory failure refractory to heliox, racemic epinephrine, and multiple bronchoscopic dilations. She was intubated and scheduled for surgery. Although the patient’s morbid obesity and long-segment stenosis were high-risk factors for tracheal resection, the patient was not diabetic and had adequate nutritional reserve, with a pre-operative albumin level of 3.7 g/dL. Surgical resection was performed. Through transcervical exposure, rings 3–7 were removed. Montgomery, subcarinal, and right hilar release maneuvers were performed. The anastomosis was constructed with interrupted polydioxanone and Vicryl suture and was buttressed anteriorly with a sternohyoid flap. A guardian stitch was placed using #1 Prolene suture to maintain neck flexion.

The patient was extubated 13 hours postoperatively. Protection measures were taken including diligent oral and incision hygiene, head-of-bed elevation, antiemetic and antitussive medication administration. Unfortunately, the patient developed delirium as well as febrile seizures (which she had experienced previously), and she required reintubation on postoperative day (POD) 6 due to inability to protect her airway. On POD 8, she developed massive subcutaneous emphysema prompting emergency bronchoscopy, which demonstrated membranous dehiscence. She underwent immediate neck re-exploration, anastomotic revision, and airway closure over a Hood 14 mm T-tube. Bilateral sternocleidomastoid muscle flaps buttressed the airway and T-tube. Three days later, she developed subcutaneous emphysema. On bronchoscopy, there was complete airway separation. Venovenous extracorporeal membrane oxygenation was initiated. Repeat neck exploration was performed, cross table ventilation initiated, and the trachea was left in discontinuity. Two days later (13 days after the initial resection), airway reconstruction using a soft tissue free flap was attempted with assistance from otorhinolaryngology. A right forearm free flap was harvested but thrombosed prior to vessel ligation. A left arm flap was not harvested due to extensive ecchymoses from intravenous and arterial line placements. A right deltoid flap was harvested but was too large to fit into the neck surgical bed.

**Figure 1:**
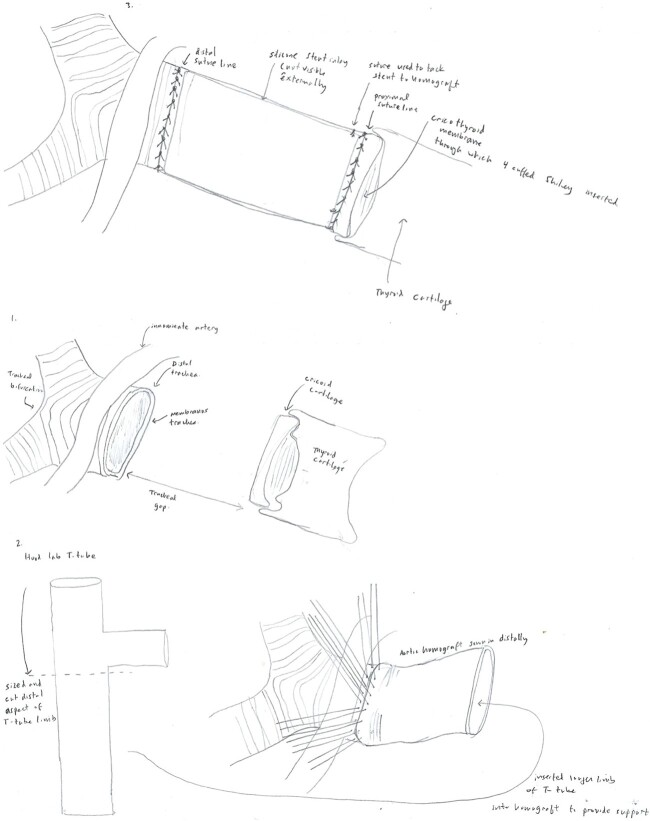
Diagram displaying the anatomy of the patient’s dehiscence at the time of her tracheal replacement using the aortic allograft (1), and the method of stent and homograft placement (2–3).

A 14 mm × 80 mm cryopreserved aortic graft, internally supported with a Hood 18 mm T-tube silicone stent was selected as a salvage replacement option. The distal anastomosis was performed with interrupted 3-0 Prolene sutures. A 4-Shiley cuffed tracheostomy tube was placed through the cricothyroid membrane, and the proximal anastomosis was performed with interrupted 3-0 Prolene sutures (Fig. 1). A segment of the deltoid flap was used to provide separation between the innominate artery and anterior anastomosis. Postoperatively, bronchoscopies were performed twice daily. ECMO was weaned after 6 days. During her admission, she required neck washouts, wound vacuum placement, and gastrojejunal tube placement. She was discharged to a long-term care facility after 70 hospital days before eventual discharge home. The patient’s postoperative course has been complicated by periods of non-adherence with home respiratory care, suicide attempts, and self-decannulation of her tracheostomy.

**Figure 2:**
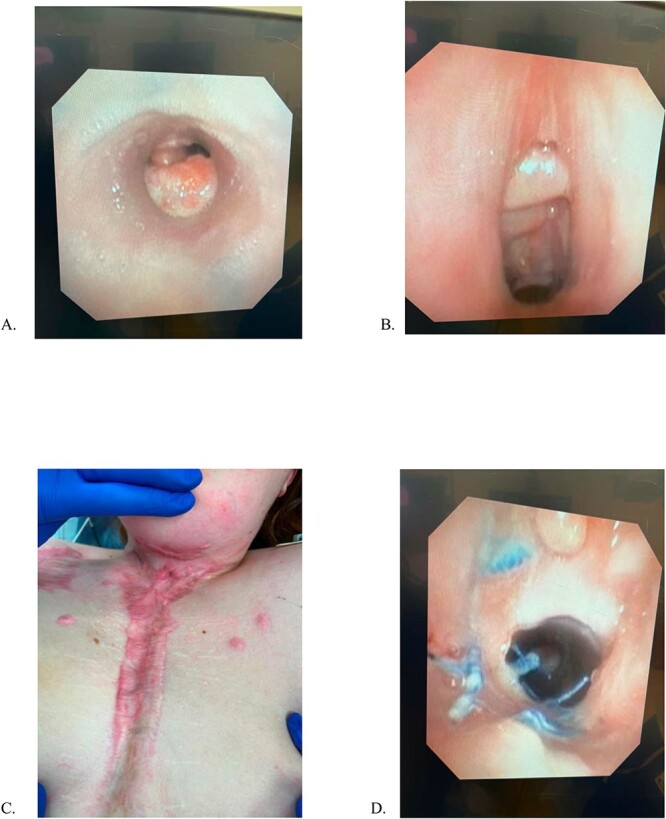
(A) Mid-tracheal stent, (B) Subglottic space, previous tracheostomy site, (C) Sternal incision, (D) Proximal Anastomosis. All images were obtained 5/6/23, 15 months after aortic homograft reconstruction.

She remains closely followed in thoracic surgery and psychiatry clinics and undergoes monthly surveillance bronchoscopies (Fig. 2). She remains home, functioning independently without oxygen or tracheostomy support. At 24 months postoperatively, she remains the longest documented survivor with a completely circumferential tracheal resection with intact aortic homograft.

## Discussion

The first tracheal replacement using cadaveric aortic allograft was described in a patient who experienced tracheal anastomotic dehiscence after heart and lung transplant [5]. No stent was placed, and the allograft was removed during repeat transplant 3 days later. Wurtz et al. reported using either fresh or cryopreserved aortic allograft for tracheal replacement for tracheal malignancies, with 4 of 6 patients alive at mean follow-up time of 34 months [6]. Martinod et al. tissue engineered aortic grafts at −80°C to regenerate cartilaginous and epithelial tissue in the neotrachea for two patients with intact native membranous tracheas, both of whom were breathing without tracheostomy support at maximal follow-up time of 67 months [7].

The use of cadaveric aorta for tracheal replacement has three principal advantages: availability, no need for immunosuppression, and ability to cover long tissue gaps [4]. Disadvantages include graft pliability, lack of intrinsic mucociliary clearance, and increased infection risk [8]. Intraluminal stenting appears useful for the maintenance of graft patency, but these stents can be a source of infection, granuloma formation, and mucus plugging. Other options for replacement including tracheal transplantation and tissue-engineered trachea have been implemented with varying degrees of success, but these methods require significant preparation time to ensure graft revascularization, which was not compatible with the urgency of our case [1, 9].

We demonstrate a feasible option for challenging cases of anastomotic dehiscence after tracheal resection. Several aspects warrant further investigation: long-term need for tracheostomy, optimal aortic graft preservation strategy, surgical technique, and stent type.
